# Safety outcomes of pembrolizumab with platinum agent chemotherapy combined with 5-fluorouracil or taxane derivative in head and neck cancer

**DOI:** 10.1177/10781552231217686

**Published:** 2023-12-03

**Authors:** Benjamin Lee, Sarah S Chehab, Wenyi Fan, Michael J Schell, Kedar S Kirtane, Anand B Shah

**Affiliations:** 1 Department of Pharmacy, 25301 Moffitt Cancer Center, Tampa, FL, USA; 2 Department of Biostatistics and Bioinformatics, 25301 Moffitt Cancer Center, Tampa, FL, USA; 3 Department of Head and Neck-Endocrine Oncology, 25301 Moffitt Cancer Center, Tampa, FL, USA

**Keywords:** Metastatic head and neck cancer, oral cancer, chemoimmunotherapy, immunotherapy

## Abstract

**Introduction:**

For patients with metastatic head and neck squamous cell cancer (HNSCC), the outcomes of pembrolizumab in combination with a platinum agent and taxane as first-line therapy remain unknown. The purpose of this study is to characterize the impact of substituting the 5-fluorouracil (5-FU) backbone for a taxane in this chemoimmunotherapy regimen on safety/tolerability and survival outcomes.

**Methods:**

This was an IRB-approved, single-center, retrospective, active comparator, new-user design study in adult patients with HNSCC treated between January 2018 and September 2021. The primary objective was to assess safety and tolerability of pembrolizumab in combination with a platinum agent and taxane against an active comparator arm of pembrolizumab in combination with a platinum agent and 5-FU. Safety and tolerability were evaluated by assessing differences in overall toxicities, with further secondary analysis evaluating differences in hematologic toxicities and pre-defined non-hematologic toxicities.

**Results:**

There was no statistical difference demonstrated with the primary endpoint between the cohorts. Reduced toxicity rates were found in the taxane arm for mucositis and creatinine levels. No grade 4 non-hematologic toxicities were reported. Patients receiving 5-FU were more likely to have dose reductions upfront, discontinue treatment due to intolerances and had significantly higher mucositis.

**Conclusions:**

This study helps to characterize the safety profile and activity of pembrolizumab in combination with a platinum agent and taxane derivative in HNSCC patients. Within our study, substitution of 5-FU with a taxane did not show an increased risk of toxicities, worsened survival, or decreased odds of achieving a response. Mucositis and elevated creatinine rates were significantly reduced within the taxane arm.

## Introduction

Head and neck cancers encompass all tumors originating from the oral cavity, larynx, pharynx, paranasal sinuses, salivary glands, and mucosa with squamous cell carcinoma histology occurring in over 90% of cases.^1^ Distant metastases are less common at initial presentation; however, more advanced cancers are also associated with worse survival.^1^ Pembrolizumab is an programmed cell death protein-1 (PD-1) antibody which was recently approved by the FDA in the first-line setting for the treatment of metastatic or unresectable, recurrent head and neck squamous cell carcinoma (HNSCC) in combination with a platinum agent and 5-fluorouracil (5-FU).^2,3^

This approval was based on the KEYNOTE-048 trial in which pembrolizumab plus 5-FU based chemotherapy was compared to cetuximab plus 5-FU based chemotherapy, the previous standard treatment for metastatic HNSCC.^2^ Patients in the pembrolizumab group received pembrolizumab 200 mg, cisplatin 100 mg/m^2^ or carboplatin area-under-the-curve 5 mg/m^2^ and 5-FU 1000 mg/m^2^ continuous infusion per day for four consecutive days every three weeks for six cycles with pembrolizumab continuing for up to 35 cycles.^1^ This combination demonstrated improved overall survival (OS), longer duration of response, and comparable safety profile compared to standard of care.^2^ As a result, the combination of pembrolizumab, platinum agent, and 5-FU is an National Comprehensive Cancer Network (NCCN) category 1 preferred first-line regimen.^1^

Substituting 5-FU with a taxane-based regimen may provide a favorable safety profile, improved patient convenience due to the lack of a 4-day continuous intravenous infusion (CIVI) requirement, and decreased need for central venous access. Taxane substitution has been studied in the E1395 study in combination with cisplatin.^4^ In E1395, the substitution of paclitaxel in combination with cisplatin on day 1 did not show a significant difference in OS compared with 5-FU continuous infusion on days 1 through 4 and cisplatin on day 1.^4^ Toxicity was similar between both arms but gastrointestinal and hematologic toxicities were more common with the combination of cisplatin and 5-FU.^4^ Cisplatin and paclitaxel's similar efficacy and side effect profile with the added patient convenience of a one day infusion has supported the practice of substituting a taxane for 5-FU continuous IV infusion. Consequently, this practice has also translated to substituting a taxane for 5-FU in the chemoimmunotherapy regimen studied in KEYNOTE-048. This combination has not been studied in this population and thus is not FDA-approved and remains an NCCN category 2B “Other Recommended” first-line regimen.^1^

However, this combination has shown benefit in another squamous cell carcinoma patient population within non-small cell lung cancer (NSCLC) and resulted in a significantly longer OS, PFS with no significant increase in grade 3 adverse events.^5^ Based on these results in NSCLC, this combination of pembrolizumab with a platinum agent (carboplatin) and taxane (paclitaxel) is an NCCN category 1 first-line preferred option.^6^ Within metastatic HNSCC, the outcomes of pembrolizumab in combination with a platinum agent and taxane as first-line therapy remain unknown. The purpose of this study is to attempt to characterize the potential benefit of this combination and to add to the body of literature for this patient population by assessing the impact of substituting the 5-FU backbone for a taxane in this chemoimmunotherapy regimen on safety/tolerability and survival outcomes.

## Patients and methods

### Study design

This was an IRB-approved, single-center, retrospective, active comparator, new-user design study in adult patients with HNSCC who were treated between January 2018 and September 2021. A waiver of consent was granted. The primary objective was to assess safety and tolerability of pembrolizumab in combination with a platinum agent and taxane derivative against an active comparator arm of pembrolizumab in combination with a platinum agent and 5-FU. Safety and tolerability were evaluated by assessing differences in overall toxicities, which included hematologic and pre-determined nonhematologic toxicities, with further secondary analysis evaluating differences in hematologic toxicities and pre-defined non-hematologic toxicities. Total number of cycles administered, dose reductions, treatment delays, missed doses, and subsequent therapies were also collected. Pre-defined non-hematologic toxicities were mucositis, nausea, vomiting, diarrhea, fatigue, serum creatinine increase, peripheral neuropathy, and palmar-plantar erythrodyesthesia syndrome. Toxicity data were collected through chart review using Common Terminology Criteria for Adverse Events, version 5.0, to assess for severity of toxicities. The secondary objectives were to assess objective response rates (ORR), time to tumor progression (TTP), progression-free survival (PFS), and OS. ORR was determined by response at first scan after at least two cycles of chemoimmunotherapy as documented in patient clinical record.

### Patient population

Patients were eligible for study inclusion if they were 18 years of age or older and received treatment with pembrolizumab in combination with a platinum agent (carboplatin or cisplatin) and either 5-FU or a taxane derivative (paclitaxel or docetaxel) for metastatic or recurrent HNSCC. 5-FU was administered via CIVI over 96 h with patients returning for pump disconnect after infusion completion. Patients were excluded if they received treatment indicated for a non-HNSCC diagnosis. Patient lists were abstracted from Cerner using Head and Neck Cancer-related ICD-10 codes.

### Statistical analysis

The primary safety analysis included all patients who received at least two cycles of treatment. Survival and response analysis included all patients who received at least one cycle. Overall toxicity rates were obtained by taking the worst grade of any toxicity from each patient in both cohorts. A Fisher exact test was used for 2 × 2 contingency tables, and ordinal toxicity rate data was analyzed via Mantel-Haenszel Chi-square trend tests, utilizing a two-sided alpha of 0.05. ORR was analyzed via multinomial logistic regression model utilizing the patient's response at first scan after receiving at least two cycles of their combination therapy. PFS and OS were analyzed via the Kaplan-Meier method.

## Results

Sixty-six patients (*N* = 66) treated between January 2018 and September 2021 were screened, with 12 patients being excluded. Fifty-four patients were included with 35 patients receiving a pembrolizumab, platinum agent, and taxane derivative combination, and 19 patients receiving a pembrolizumab, platinum agent, and 5-FU combination. Two patients receiving the taxane derivative combination only received one cycle of treatment and were excluded from the safety analysis. Baseline characteristics of the 54 patients included are outlined in Table 1. A higher proportion of patients in the 5-FU cohort had an Eastern Cooperative Oncology Group (ECOG) performance status (PS) of 0 (58% vs 29%), received prior therapy for metastatic or recurrent HNSCC (68% vs 34%), and were more likely to have received cisplatin as their platinum agent (79% vs 0%). Common sites of metastases included lungs (*N* = 30), lymph nodes (*N* = 10), and bone (*N* = 8). Around 35% (*N* = 19) of all patients in both cohorts received at least one prior line of therapy for metastatic or recurrent HNSCC. With regards to PDL1 expression, patients in the taxane cohort had a higher prevalence of CPS ≥ 20 (40% vs 26%). Data for PDL1 expression was unknown or not available for 26% of patients in both cohorts.

**Table 1.** Patient baseline characteristics.

	Total	Taxane	5-FU	*p*-value
Total, *N* (%)	54 (100)	35 (65)	19 (35)	
Age, *N* (range), years	64 (20–86)	62.6 (20–86)	63.9 (36–86)	0.556
Male, *N* (%)	45 (83)	29 (83)	16 (84)	>0.999
HPV positive, *N* (%)	24 (44)	14 (40)	10 (53)	0.622
ECOG performance status, *N* (%)				0.071
0	21 (39)	10 (29)	11 (58)	
1	25 (46)	19 (54)	6 (31)	
2	8 (15)	6 (17)	2 (11)	
Platinum agent, *N* (%)				<0.001
Cisplatin	15 (28)	0 (0)	15 (79)	
Carboplatin	39 (72)	35 (100)	4 (21)	
Received prior therapy, *N* (%)	25 (46)	12 (34)	13 (68)	0.016
Number of prior therapies				
0	29	23	6	
1	19	9	9	
2	3	1	3	
3+	3	2	1	
PDL1 expression				0.223
CPS 0	7 (13)	6 (17)	1 (5)	
CPS 1–19	14 (26)19	6 (17)	8 (42)	
CPS ≥ 20	(35)	14 (40)	5 (26)	
Unknown/Not recorded	14 (26)	9 (26)	5 (26)	

There was no statistical difference demonstrated with the primary endpoint between the cohorts. Overall toxicity rates by overall grade, hematologic or non-hematologic did not differ by cohort (Table 2). There were no grade 4 non-hematologic toxicities reported in either treatment cohort.

**Table 2.** Total number of toxicities and grade by treatment cohort.

	Taxane (*N* = 33)	5-FU (*N* = 19)	*p*-value
Neutropenia			0.424
Grade 1-2	4 (12%)	4 (22%)	
Grade 3+	8 (24%)	2 (11%)	
Anemia			0.548
Grade 1-2	22 (67%)	9 (47%)	
Grade 3+	9 (27%)	6 (32%)	
Thrombocytopenia			0.193
Grade 1-2	6 (18%)	3 (16%)	
Grade 3+	1 (3%)	3 (16%)	
Diarrhea	5 (15%)	1 (5%)	0.397
Nausea/Vomiting	10 (30%)	5 (26%)	1.000
Mucositis			0.037
Grade 1-2	2 (6%)	1 (5%)	
Grade 3+	0 (0%)	3 (16%)	
Peripheral neuropathy			0.305
Grade 1-2	12 (36%)	6 (32%)	
Grade 3+	2 (6%)	0 (0%)	
Creatinine increase	0 (0%)	3 (16%)	0.044
Infusion related reaction	2 (6%)	1 (5%)	1.000
Immune-related adverse event			0.126
Grade 1-2	6 (18%)	1 (5%)	
Grade 3+	1 (3%)	0 (0%)	

Mucositis (*p* = 0.037) and creatinine (*p* = 0.044) were found to be statistically significantly higher in the 5-FU cohort, with no other statistically significant differences in overall toxicities and grade of overall toxicities (Table 2). There were also numerical higher levels of grade 3 or higher thrombocytopenia (16% vs 3%) in the 5-FU cohort, though not statistically significant. Patients in the taxane cohort had numerically higher rates of diarrhea (15% vs 5%), and grade 3 or higher neutropenia (24% vs 11%), though this result was not statistically significant.

Patients in the 5-FU cohort were also found to have an increased prevalence of initial 5-FU dose reductions compared to the every-3-week taxane cohort (70% vs 7%). There was a higher rate of dose reductions required during any cycle for patients in the 5-FU cohort compared to the taxane cohort (42% vs 18%). These differences were not statistically significant. The most common reason for dose reduction in all patients was due to hematologic toxicities.

There was a total of eight (24%) treatment delays in the taxane cohort compared to six (32%) in the 5-FU cohort. Reasons for treatment delay in the taxane cohort included three instances due to scheduling conflicts (all from one patient), two instances due to hematologic toxicity, one instance due to concurrent palliative radiation, one instance due to infection, and one instance due to placement of a palliative G-tube. Treatment delay occurrences in the 5-FU cohort included four due to concurrent hematologic toxicity.

At the time of data cut-off, 84% of patients receiving 5-FU had discontinued treatment compared to 80% in the taxane cohort. Both cohorts had same median number of cycles (four cycles). The most common reason for discontinuation in both cohorts was progression of disease (48% for taxane cohort vs 42% for 5-FU cohort). More patients in the 5-FU cohort discontinued treatment due to intolerance (26% vs 3%).

**Figure 1.**
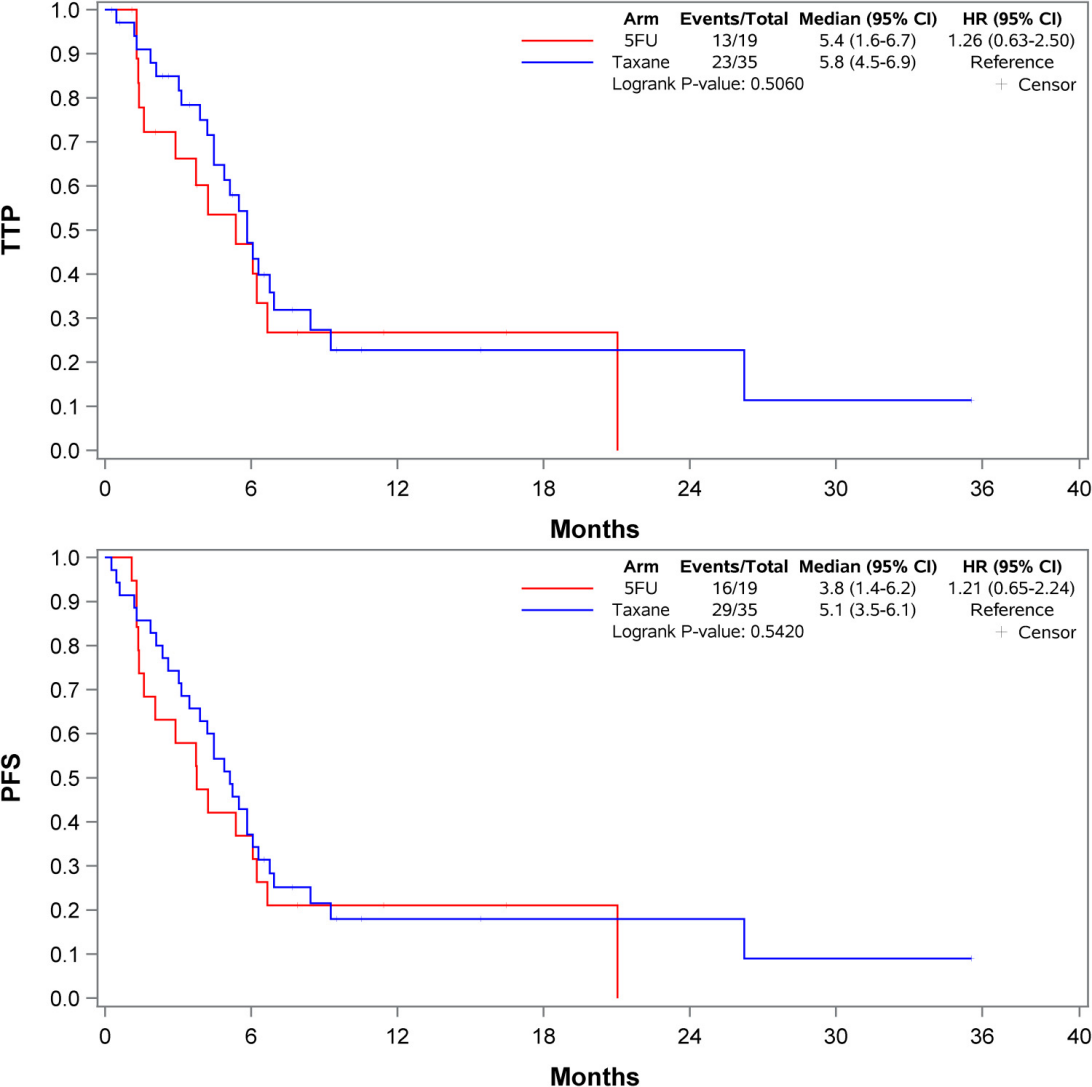
Kaplan-Meier curve for time to tumor progression and progression free survival.

Although this study was not powered for the secondary endpoints of ORR, TTP, PFS, and OS, there were no statistically significant differences found between the cohorts (Figure 1). Within the taxane cohort, 43% of patients achieved a partial or complete response and 31% had stable disease compared to 32% and 21% within the 5-FU cohort, respectively. However, there was no statistically significant difference in overall response rate at first scan (*p* = 0.24). A multinomial logistic regression was also performed to assess if first-line treatment was predictive of response at first scan and failed to demonstrate a difference in response between first-line treatment and subsequent line treatment. Patients in the taxane cohort had a median PFS of 5.1 months compared to 3.8 months in the 5-FU cohort (hazard ratio (HR) 1.21; 95% confidence interval (CI), 0.65 to 2.24; *p* = 0.54). OS was 11.4 months in the taxane cohort compared to 9.6 months in the 5-FU cohort (HR 0.76; 95% (CI), 0.37 to 1.55; *p* = 0.45).

## Discussion

Head and neck cancer has historically been associated with pain, malnutrition, overall reduced quality of life, and poor survival due to patient comorbidities and intolerance to therapy.^1^ First-line treatment for metastatic HNSCC prior to 2008 consisted primarily of single-agent and combination systemic therapy.^1,7^ The phase III randomized EXTREME trial in 2008 introduced cetuximab in combination with 5-FU and a platinum agent which demonstrated improvements in ORR and median OS leading to a standard of care for many years.^1,7^ The advent of immune checkpoint inhibitors led to the phase III randomized KEYNOTE-048 trial which supplanted the EXTREME regimen and created a new standard of care with pembrolizumab in combination with 5-FU and a platinum agent.^1,2^ Though this new standard of care demonstrated efficacy, logistical and safety challenges of a 96-h continuous 5-FU infusion has led to the practice of substituting 5-FU with a taxane derivative.^8^ The results of our study contribute to the body of literature with this combination and further characterize this substitution in the HNSCC patient population.

Use of a 96-h continuous infusion of 5-FU can be a logistical challenge to patients due to coordination of multiple appointments for pump removal, need for central venous access for administration, and potential risk for life-threatening toxicities due to dihydropyrimidine dehydrogenase deficiency.^8^ Nutritional status may also be affected as 5-FU is associated with toxicities such as grade 3 or 4 mucositis in 13% of patients.^8^ This rate of grade 3 or 4 mucositis is similar to the 16% seen in our study. Therefore, a taxane derivative can be beneficial to patients in offering a more convenient treatment as a one-day infusion along with a favorable safety profile reducing the risk of severe mucositis.^4,8^

The substitution of 5-FU with a taxane derivative did not demonstrate a statistically significant difference with regards to the primary endpoint of overall toxicities along with the secondary endpoints of ORR, PFS, and OS. However, this study was also not powered for the secondary endpoints. Numerical differences were noted when evaluating individual toxicities. The 5-FU cohort experienced statistically significantly higher rates of creatinine increases, grade 3 or higher mucositis and thrombocytopenia while the taxane cohort experienced numerically higher rates of diarrhea, and grade 3 or higher peripheral neuropathy and neutropenia though these results were not statistically significant. This toxicity profile contrasts with the E1395 study in which the 5-FU/cisplatin arm demonstrated higher rates of hematologic toxicities and diarrhea.^4^ This difference in toxicity profiles with regards to hematologic toxicities can be explained by all patients in the taxane cohort receiving carboplatin which has been shown to be more myelosuppressive than cisplatin.^9^ The total number of toxicities within the 5-FU cohort may have also been impacted due to the majority of patients receiving a dose reduction upfront compared to the taxane cohort.

Several limitations are inherent to the design of the study such as selection bias. Selection bias is a risk because the study population is not guaranteed to be representative of the general population; however, our patient population was similar to reported patient epidemiologic factors in HNSCC along with in baseline characteristics in KEYNOTE-048 with regards to age, sex, ECOG PS, and the majority of our patients being CPS ≥ 1.^2,10^ Some data results were limited due to the retrospective nature of the study and reliance on physician documentation in the electronic medical record. Due to this study being a single-center experience, this study should be implemented with caution due to potential differing institution protocol and supportive care standards. Our study also included patients receiving treatment in the subsequent line setting. Strengths of this study do include the exclusion of non-squamous cell head and neck cancer histology along with utilization of a standard of care active comparator cohort.

In conclusion, this single-center, real-world experience of substituting 5-FU with a taxane derivative demonstrates this combination as a viable and potentially safer option for treatment of metastatic or recurrent HNSCC due to reduced toxicities. This study helps to characterize the safety profile and activity of pembrolizumab in combination with a platinum agent and taxane derivative compared to 5-FU and a platinum agent in HNSCC patients. Within our study, substitution of 5-FU with a taxane did not show an increased risk of toxicities, worsened survival, or decreased odds of achieving a response. Moreover, substitution of 5-FU with a taxane showed a statistically significant decrease in mucositis rates and elevated creatinine levels. The median PFS (3.8 months vs 5.1 months) and median OS (9.6 months vs 11.4 months) of 5-FU patients were lower compared to those seen in the landmark KEYNOTE-048 trial but this can also serve as an indicator to the potential challenges of this regimen outside of a clinical trial setting.^2^ Further prospective randomized-control trials are needed to confirm this option as viable and safer; however, in the absence of other data this paper may assist clinicians in determining which patients with HNSCC may not be suitable for 5-FU continuous infusions. Furthermore, an ongoing phase 4, single arm study hopes to further characterize this combination in the first-line setting though completion is not expected until mid-2024.^11^
